# Do health technology assessments comply with QUOROM diagram guidance? An empirical study

**DOI:** 10.1186/1471-2288-7-49

**Published:** 2007-11-20

**Authors:** Daniel Hind, Andrew Booth

**Affiliations:** 1 School of Health and Related Research (ScHARR), University of Sheffield, Regent Court, 30 Regent Street, Sheffield, S1 4DA, UK

## Abstract

**Background:**

The Quality of Reporting of Meta-analyses (QUOROM) statement provides guidance for improving the quality of reporting of systematic reviews and meta-analyses. To make the process of study selection transparent it recommends "a flow diagram providing information about the number of RCTs identified, included, and excluded and the reasons for excluding them". We undertook an empirical study to identify the extent of compliance in the UK Health Technology Assessment (HTA) programme.

**Methods:**

We searched Medline to retrieve all systematic reviews of therapeutic interventions in the HTA monograph series published from 2001 to 2005. Two researchers recorded whether each study contained a meta-analysis of controlled trials, whether a QUOROM flow diagram was presented and, if so, whether it expressed the relationship between the number of citations and the number of studies. We used Cohen's kappa to test inter-rater reliability.

**Results:**

87 systematic reviews were retrieved. There was good and excellent inter-rater reliability for, respectively, whether a review contained a meta-analysis and whether each diagram contained a citation-to-study relationship. 49% of systematic reviews used a study selection flow diagram. When only systematic reviews containing a meta-analysis were analysed, compliance was only 32%. Only 20 studies (23% of all systematic reviews; 43% of those having a study selection diagram) had a diagram which expressed the relationship between citations and studies.

**Conclusion:**

Compliance with the recommendations of the QUOROM statement is not universal in systematic reviews or meta-analyses. Flow diagrams make the conduct of study selection transparent only if the relationship between citations and studies is clearly expressed. Reviewers should understand what they are counting: citations, papers, studies and trials are fundamentally different concepts which should not be confused in a diagram.

## Background

With an estimated seven systematic reviews being published every day [1], there is an increasing concern with the quality of reporting. Recognised standards exist for reporting meta-analyses of trials and observational studies [2,3] However there are no standards for systematic reviews in general (see terminology box for the distinction between systematic reviews, health technology assessments and meta-analyses). As a result, peer-reviewed publications frequently demand that standards devised specifically for reporting meta-analyses should inform the reporting of systematic reviews more generally. The Quality of Reporting of Meta-analyses (QUOROM) statement provides guidance for improving the quality of reporting of meta-analyses of randomised controlled trials (RCTs). Its authors state that the QUOROM checklist, "might also be useful for reporting of systematic reviews" (without statistical aggregation) and some medical journals (among them, *BMJ *and *BMC Medicine*) now incentivize its use in systematic reviews, even where there is no meta-analysis, by making compliance a pre-condition of publication. Amongst other things, the QUOROM statement attempts to make the process of study selection transparent, reproducible and systematic: "Authors are asked to provide a flow diagram providing information about *the number of RCTs identified*, included, and excluded and the reasons for excluding them" (our emphasis) [2].

### *Terminology Box*

#### *Systematic review*

*"A review of a clearly formulated question that uses systematic and explicit methods to identify, select, and critically appraise relevant research, and to collect and analyse data from relevant primary studies. Statistical techniques (meta-analysis) may or may not be used to summarise the results of the included studies" *[4].

#### *Meta-analysis*

*The use of statistical techniques to integrate the results of primary studies (usually randomised controlled trials) in order to obtain a more precise estimate of clinical effect. Sometimes misused as a synonym for systematic reviews, where the review includes a meta-analysis*[4]. *A meta-analysis may be published without a systematic review or health technology assessment, most frequently when two or more trialists investigating the same intervention combine their data.*

#### *Health Technology Assessment (HTA)*

*A process of evidence review and synthesis to support policy makers. HTAs are sometimes distinguished from systematic reviews, by the following characteristics: (1) their questions, methods and the evidence they consider are driven by the interests of policy-makers rather than those of clinicians or researchers; (2) they are inter-disciplinary efforts (most include cost-effectiveness analyses, unlike systematic reviews which are mainly confined to analyses of clinical effectiveness); (3) their results tend to be disseminated outside of the clinical and research communities*[5,6]. *Some, but not all HTAs, incorporate systematic reviews and/or meta-analyses.*

**Figure 1:**
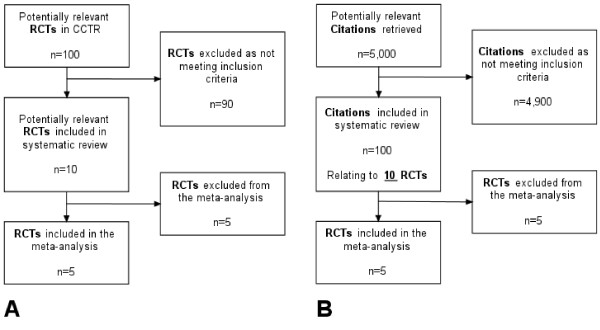
Idealised (A) and pragmatic (B) logical study selection diagrams.

This statement assumes that randomised controlled trials (RCTs) are the only types of primary study to be included in a systematic review and that it is the *study*, not the *publication *or *citation *that is the preferred unit of analysis. In its simplest form, the study selection diagram would report how many *studies *are retrieved, excluded and included: the diagram would start by counting *studies *at the top and conclude by counting *studies *at the bottom (Figure 1A). Diagrams recording only studies (as opposed to citations or publications) might be produced under two conditions: (a) where a single database is used and where each record in that database relates to a study *per se*, rather than to a citation for a report from that research study; and, (b) where researchers know the relationship between bibliographic citations retrieved by the searches and the number of research studies they represent. For at least three reasons, neither of these conditions usually pertains in a systematic review or health technology assessment.

First, most literature searches use bibliographic databases in which each record is a *citation *with a one-to-one relationship to a *publication*, not a *study*. Second, not all *publications *relate to research *studies*; many are editorials, letters and opinion pieces. Third, there is often not a one-to-one relationship between *studies *and *publications*: most randomised controlled trials will report at multiple follow-up intervals; many will issue multiple publications at the same time point. As a result, it is common for reviewers to have to disentangle the relationship between the numbers of *citations*, *papers *and *studies *at some point when reporting study selection. However, given that the number of citations retrieved frequently runs into thousands, establishing the relationship between the total numbers of *citations *retrieved and the *studies *they represent would be laborious, expensive, of questionable value and, in fact, is rarely undertaken. In summary, the reader cannot assume a simple one-to-one relationship between *citations *and *publications*, and there is rarely, if ever, a one-to-one relationship between either of these and primary research *studies*.

As a result, systematic reviewers tend to start the study selection diagram with the number of bibliographic *citations *(not studies) as the unit of analysis, usually (but not invariably) excluding duplicate citations from different bibliographic databases. However, most reviewers still aim to complete the study selection diagram with the number of *studies *(not citations) included in the review or meta-analysis. The authors of this study consider that, compliance with the QUOROM statement, requires that reviewers make explicit the relationship between the number of *citations *and the number of *studies *at some later stage in the study selection diagram (as in Figure 1B). However, this is not widespread practice.

To assess compliance with the study selection diagram requirement of the QUOROM statement, we undertook an empirical study of its use in systematic reviews published between 2001 and 2005 within the UK NHS Health Technology Assessment (HTA) programme. The HTA programme prioritises and funds research projects, of which about 20 to 30 per year are systematic reviews of the published literature on clinical effectiveness and methodological topics. There were two primary objectives: (1) to record uptake of QUOROM study selection diagrams in, (a) systematic reviews with meta-analyses; (b) all systematic reviews; and, (2) to record how many studies establish the relationship between citations and studies within a QUOROM study selection diagram.

## Methods

In May 2006, we searched Medline for the HTA programme's monographs ("health technology assessment winchester england.jn") published between 2001 and 2005. Where citations had the phrase "systematic review" in either the title or the abstract, we retrieved the monograph and confirmed that it was a systematic review of the published literature on the clinical effectiveness of a therapeutic intervention. Reviews of diagnostic and screening technologies, qualitative research, methodological aspects, service delivery or behavioural topics were excluded.

One researcher (DH) examined the main body and appendices of all reports, recording whether each study contained a meta-analysis of controlled trials (excluding those presented as examples in methodological studies), whether a QUOROM study selection diagram was presented and, if so, whether it expressed the relationship between the number of citations and the number of studies. Another researcher (AB) extracted (blinded) the same variables from a 20% sample (n = 19) of all eligible studies.

Because, strictly speaking, the QUOROM guidelines refer to the quality of reporting of *meta-analyses *(rather than all systematic reviews), we performed separate compliance analyses, both on all systematic reviews and on the subset of those containing a meta-analysis of randomised controlled trials. These analyses would thus measure compliance with the implicit and explicit intent of the QUOROM guidelines. We used Cohen's κ to test inter-rater reliability for whether or not a review contained a meta-analysis and whether or not a study selection diagram contained a citation-to-study relationship [7].

## Results

**Figure 2:**
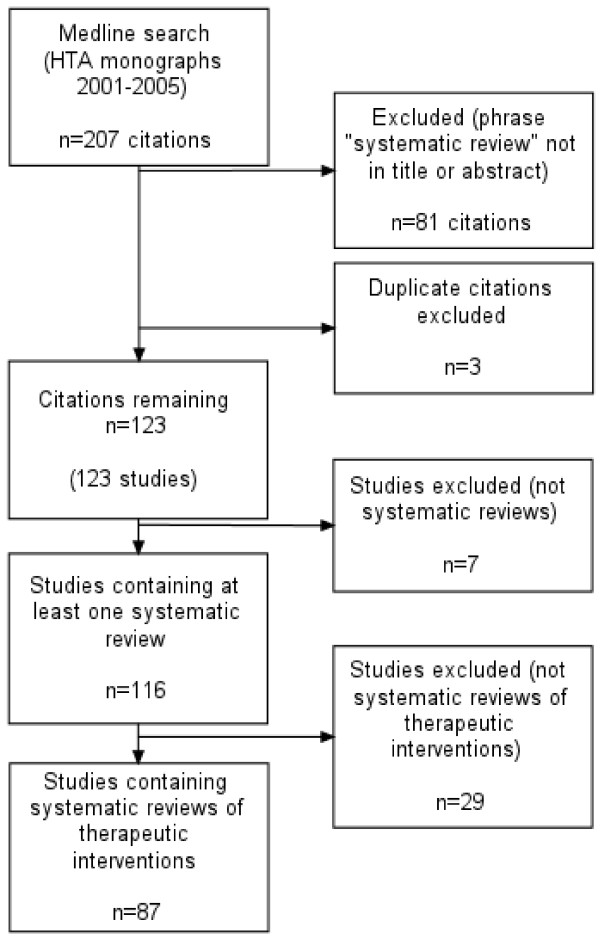
Study selection for empirical study of QUOROM compliance.

The initial search retrieved 207 citations of which 108 had the phrase "systematic review" in the title and a further 18 in the abstract (126 in total). Three duplicates were excluded leaving 123 monographs. Seven monographs were excluded because they did not contain a systematic review. Figure 2 shows the study selection process.

Of the remaining 116 studies, all claiming to be a systematic review (either in the title or text), we included only 87 which evaluated the clinical effectiveness of therapeutic interventions. We excluded 12 diagnostic and screening studies, 11 methods studies, three qualitative studies, two service delivery studies, and one behavioural study (total 29).

There was good (*κ *= 0.78) and excellent (*κ *= 1) inter-rater reliability for, respectively, whether or not a review contained a meta-analysis and whether or not a study selection diagram contained a citation-to-study relationship.

Overall, 49% of systematic reviews of clinical effectiveness illustrated the study selection process with a diagram. The percentage of compliant reviews had almost doubled between 2002 and 2004 (from 36% to 68%), but declined in 2005. Where only systematic reviews containing a meta-analysis of randomised controlled trials were analysed, compliance was consistently worse than for all systematic reviews. Overall, 32% illustrated the study selection process with a flow diagram. Compliance rose from 14% to 56% between 2001 and 2004 but declined in 2005 (Table 1).

**Table 1:** Effectiveness reviews illustrating study selection in a flow diagram

**All systematic reviews of therapeutic interventions: any flow diagram**				
	**N reviews**	**n flow diagrams (%)**	**Citation to study relationship (% of all diagrams)**	**Citation to study relationship: % of all reviews**

2001	18	7 (38.9)	2 (28.6)	11.1
2002	22	8 (36.4)	5 (62.5)	22.7
2003	15	8 (53.3)	3 (37.5)	20.0
2004	19	13 (68.4)	6 (46.2)	31.6
2005	13	7 (53.8)	4 (57.1)	30.8
**2001–05**	**87**	**43 (49.4)**	**20 (46.5)**	**23.0**

**Systematic reviews containing a meta-analysis: any flow diagram**				

	**N reviews**	**n flow diagrams (%)**		
		
2001	7	1 (14.3)		
2002	10	2 (20)		
2003	5	1 (20)		
2004	9	5 (55.6)		
2005	7	3 (42.9)		
**2001–05**	**38**	**12 (31.6)**		

Only 20 studies (23% of all systematic reviews; 43% of those having a study selection diagram), had a diagram which expressed the relationship between citations and studies at any point. Of those including a diagram, expression of the citation to study relationship improved from 29% (2 of 7) in 2001 to 57% (4 of 7) in 2005 (Table 1).

## Discussion

### Principal findings

Although the QUOROM statement recommends inclusion of a study selection flow diagram, our study shows that compliance in a sample of recent systematic reviews was poor. This finding pertained regardless of whether or not the systematic reviews included a meta-analysis. While it is true that, on aggregate, compliance has improved over time; given an unexplained reduction in prevalence for the most recent year under study it is unclear whether this trend is continuing.

Furthermore, of those systematic reviews which do illustrate study selection using a flow diagram, less than half enable the reader to follow a transparent process of study selection from a known number of identified citations to a known number of studies. It is our contention that, unless this relationship is made clear, the diagram does not serve the reader well. Indeed the diagram may actually confuse readers when there are clear discrepancies in totals in different boxes.

We recommend that, in those systematic reviews where reviewers incorporate data sources in which citations do not have a direct one-to-one relationship with studies, reviewers make this relationship transparent at some point in a study selection diagram.

### Strengths and weaknesses of the study

This study sampled only a proportion of the recent output of one publication, the HTA monograph series. The results cannot be generalised to other publications. Nevertheless, many researchers working on this series are highly experienced systematic reviewers and the standard of the peer review at the HTA programme is considered to be high. Therefore there is probably sufficient information in this study to hypothesize that compliance with the QUOROM statement is poor, six years on.

One criticism of our study could be that might measure either awareness of, or compliance with reporting standards on the part of individual researchers or awareness and enforcement of reporting guidelines on the part of serial editors. Following the completion of our analysis the NHS HTA programme confirmed that inclusion of a study flow diagram been editorial policy since February 2004. In effect then, the first three years of our sample measure the awareness of reporting standards on the part of individual researchers, whereas the last two years are measuring how successful the NHS HTA have been in enforcement. The mere existence of reporting guidelines or even a journal's endorsement of them will not necessarily translate into their use by authors, which needs to be actively enforced [8].

A further criticism of our study might be the use of the phrase "systematic review" in the title or abstract as an eligibility criterion. If this study were to be repeated in the journal literature, such a strategy might be considered unwise: only 50% of reports have this term in the title or abstract [1]. However, it has been the editorial policy of the NHS HTA programme to include the words "systematic review" in the title of monographs which incorporate a systematic review over most of our study period (since 2002) so, we believe that the risk of under-retrieval is minimal.

Our study demonstrates that the existence of reporting guidelines does not necessarily lead to improvements in the conduct of research and that there can be considerable delay in the adoption of such conventions. Similar delays have been previously reported in other audits of compliance with publication guidelines [9,10]. Compliance with such publication standards may be enhanced through the use of specific requirements at the publication end of the process such as through inclusion in Instructions to Authors.

Furthermore such standards are developed following normalisation of previously reviewed data. They may thus be subject to considerable challenge from the ingenuity and methodological innovation of researchers when faced with uncertainties regarding how such guidelines should be interpreted and implemented.

In this regard, it is important to note that we have not made the distinction between QUOROM diagram adoption by a publisher and by an individual researcher.

### Meaning of the study

Readers of systematic reviews need to be able to understand how the studies included in a systematic review have been selected. This requires that any supporting diagrams are easy to interpret and creates the need for such diagrams to share a consistent logic across reviews. Our study shows that, despite the intent of the QUOROM statement to help researchers make their work transparent to the reader, at least one component of the guidance, the study selection flow diagram, is not succeeding in this regard. It is true that a reader also has access to the textual narrative by which they can make sense of the conduct of the review. However this is unlikely to be sufficient and may carry its own problems.

### Recommendations

If the purpose of a flow diagram is to make the conduct of study selection transparent, reproducible and systematic (thereby reassuring the reader that attempts were made to minimize bias), then we believe it is useful for such diagrams to be included in any systematic review, regardless of whether statistical synthesis (meta-analysis) is undertaken. Making a reference to the relevant published reporting standards when using a flow diagram encourages dissemination and acceptance by helping the reader to understand that inclusion of the diagram is an aspect of good practice. In our sample, very few studies (even among those with a study selection diagram) actually refer directly to the QUOROM statement. Without reference to the QUOROM statement, readers who lack methodological experience will not be aware of the reason for, or the importance of the inclusion of a study selection diagram. They may also believe that the format and content of such a diagram is subject to individual interpretation, rather than representing adherence to a publishing convention. Every producer, publisher or consumer of systematic reviews needs to be aware that reporting standards exist.

Transparency in study selection also entails using consistent and transparent terminology within the narrative and study selection diagram. In particular, it is critical that the reader can distinguish whether a researcher is counting *citations*, *publications *or *studies*. The number of citations is important to researchers when conducting a literature search; the number of publications is important, after eliminating duplicates, when undertaking study selection; but, the number of studies is most important in the final analysis. For this reason, we recommend that at the earliest stage possible within the study selection diagram, researchers inform the reader how many citations remain and how many individual studies they represent (accounting for studies with multiple publications). We noted considerable variation between technology assessment reports in terms of the point at which the citation-to-study relationship is made explicit within the study selection diagram. Such variation may not in itself prove critical. It may simply reflect a pragmatic decision on the easiest point, given review workload, at which the reviewer is able to switch from citations to studies. Mapping citations to studies is certainly easier to perform towards the foot of the diagram, where few studies remain, with a few studies, than at the head, where there may be thousands of citations.

**Figure 3:**
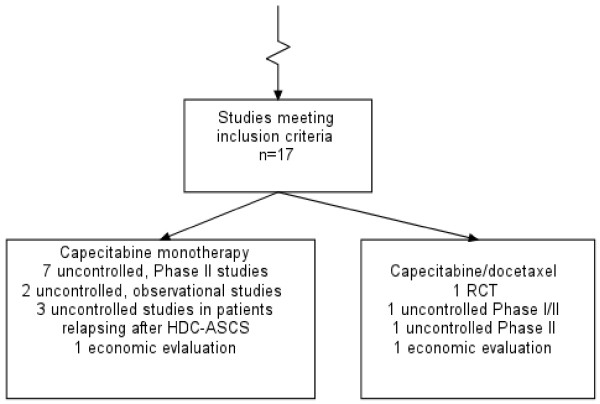
Diagram handling different review questions and study designs: from Jones L, *Health Technol Assess 2004;8(5)*.

In the monographs we reviewed, we noted that some diagrams attempted to handle multiple content variables (Figure 3). These included: (1) study-design-based variables (example: the diagram distinguishes between systematic reviews, RCTs and observational studies); (2) topic-based variables (example: references to multiple systematic reviews of effectiveness in the same diagram); and, (3) purpose-based variables (example: study selection for a systematic review of effectiveness and a review of the cost-effectiveness literature explained in the same diagram). If a single study selection diagram is used for multiple content variables, authors need to make sure that the conduct of study selection is transparent. Where a comparison of treatment effects involves the assessment of multiple outcomes, the composition of individual meta-analyses may involve different numbers of studies. Where it is impractical, or considered undesirable to present one QUOROM diagram per meta-analysis, the final box in the diagram should certainly specify the number of studies in each meta-analysis, not just that assessing the primary outcome. Clarity as to the reasons for differential composition of meta-analyses is important because systematic reviews as well as clinical trials are subject to outcome reporting bias [1].

**Figure 4:**
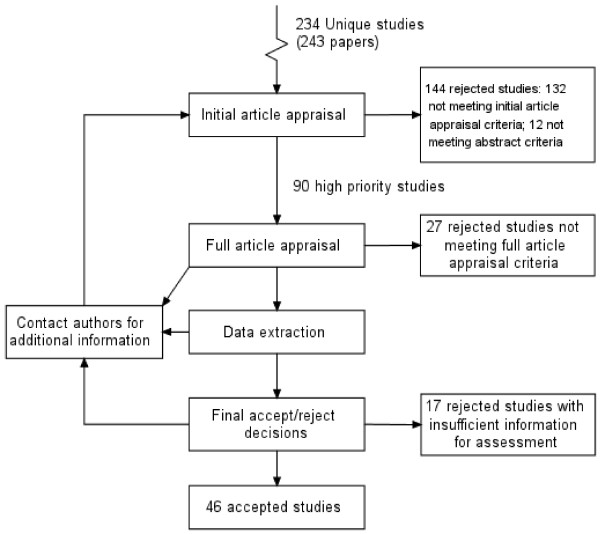
Methodological diagram: from Cooper BS, *Health Technol Assess *2003;7(39).

**Figure 5:**
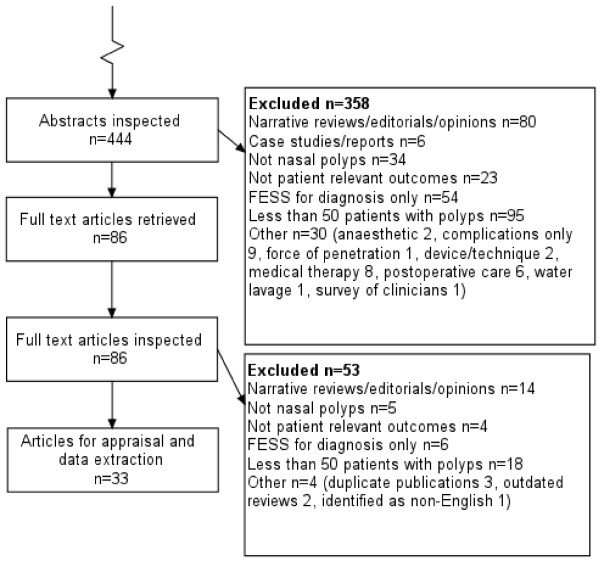
Chronological diagram: from Dalziel K, *Health Technol Assess *2003; 7(17).

We also noted three different ways of structuring a study selection flow diagram, characterised as being either methodological, chronological and logical. The *methodological type *prioritises understanding of the review process and, as a result, may have arrows feeding back between boxes and/or boxes with no numbers of citations/studies (see Figure 4). As such, they are useful in explaining the iterative nature of the systematic review process to a new researcher. The *chronological *type prioritises explanation of what the researchers did in strict chronological order: they may count exclusions made for the same reason in different boxes, and may thus demonstrate redundancy with the same number of units given in successive boxes (Figure 5: "Copies retrieved, n = 87"; "Copies inspected, n = 87"). The *logical *type (Figure 1) appears to adhere most closely to the intent of the QUOROM statement. It is retrospective; it can only be completed when the final composition of the meta-analysis is known. The inclusion or exclusion of units made for the same reason is counted in the same box regardless of source or chronology. It provides an audit trail of the *conduct *of study selection for the reader, and does not distract the reader with redundant information about process.

There is considerable variation between study selection flow diagrams in terms of the point at which papers not identified through the electronic searches (the results of handsearching or citation tracking results, grey literature and papers recommended by expert advisors) are incorporated. We recommend that these references are included in the top box, as it seems to us that this is most consistent with the logical approach to study selection flow diagram design.

Attrition of units (whether citations, papers or studies) through the study selection flow diagram needs to be transparent. Every stage of the flow diagram should have an exit box; the numbers in the exit boxes, when aggregated with the "brought forward" box should always reconcile with the total number of studies. There should not be a failure to account for studies deemed "withdrawals" (i.e. excluded by application of explicit criteria) or "dropouts" (i.e. where a full report of the study in inaccessible or unavailable) and numbers should decrease as the diagram proceeds down the page. Our study demonstrates that, for some studies at least, this is not currently the case. Just as appraisal frameworks for randomised controlled trials currently require accounting for "withdrawals" and "dropouts" so too corresponding frameworks for systematic reviews could require complete accounting for the progress of studies through the review.

### Unanswered questions and future research

A persistent dilemma for those appraising and interpreting any form of published research is the extent to which it is valid to equate quality of reporting with quality of conduct for that study. It would be valuable to establish through empirical research the extent to which a properly constituted study selection flow diagram, following a logical progression of process and fully accounting for "withdrawals" and "dropouts", might provide a reliable indicator of review quality.

## Conclusion

Compliance with the recommendations of the QUOROM statement is not universal in systematic reviews or meta-analyses. Flow diagrams make the conduct of study selection transparent only if the relationship between citations and studies is clearly expressed. Reviewers should understand what they are counting: citations, papers, studies and trials are fundamentally different concepts which should not be confused in a diagram.

Standards of reporting, such as those embodied in the QUOROM statement, seek to improve the transparency of *reporting *of methods. In so doing, they carry the implicit assumption, indeed the aspiration, that this will also improve the quality of the *conduct *of such studies (although this is yet to be established for other study designs [9,10]). The results of this study suggest that reporting guidelines may, in fact, serve an additional purpose, namely to facilitate the identification of variation and thus aid further refinement of the standards themselves. Rather than using existing standards as a methodological "stick" with which to beat deviant reviewers it is hoped that studies such as ours, through analysis of variance, may contribute to the development of standards that, at once, recognise the diversity of approaches and yet achieve the consistency and transparency that their originators intended.

## Competing interests

Both authors have worked for the NHS Health Technology Assessment programme.

## Authors' contributions

DH and AB made substantial contributions to the conception and design, acquisition of the data, analysis and interpretation of data. Both were involved in drafting the manuscript and have given final approval of the version to be published.

## Pre-publication history

The pre-publication history for this paper can be accessed here:


